# Supplementary material for: Air-Filled Bubbles Stabilized by Gold Nanoparticle/Photodynamic Dye Hybrid Structures for Theranostics
Source: Nanomaterials (Basel). 2021 Feb 6;11(2):415. doi: 10.3390/nano11020415 (PMC7915581; doi:10.3390/nano11020415)
Supplement: Supplementary file 1 [file nanomaterials-11-00415-s001.pdf]

Supplementary Materials

Air-filled bubbles stabilized by gold nanoparticle/ photodynamic dye hybrid structures for theranostics

Roman A. Barmin[1,*], Polina G. Rudakovskaya[1], Olga I. Gusliakova[2], Olga A. Sindeeva[1,2], Ekaterina S. Prikhozhdenko [2], Elizaveta A Maksimova[1], Ekaterina N. Obukhova[1], Vasiliy S. Chernyshev[1], Boris N. Khlebtsov[3], Alexander A. Solovev[4], Gleb B. Sukhorukov[1,5], Dmitry A. Gorin [1,*]

[1] Skolkovo Institute of Science and Technology, 3 Nobelya Str., 121205 Moscow, Russia; Roman.Barmin@Skoltech.ru (R.A.B.); P.Rudakovskaya@skoltech.ru (P.G.R.); O.Sindeeva@skoltech.ru (O.A.S.); Elizaveta.Maksimova@skoltech.ru (E.A.M.); E.Obukhova@skoltech.ru (E.N.O.); V.Chernyshev@skoltech.ru (V.S.C.); g.sukhorukov@skoltech.ru (G.B.S.)

[2] Remote Controlled Theranostic Systems Lab, Saratov State University, 83 Astrakhanskaya Str., 410012 Saratov, Russia; olga.gusliakova17@gmail.com (O.I.G.); prikhozhdenkoes@gmail.com (E.S.P.)

[3] Institute of Biochemistry and Physiology of Plants and Microorganisms, Russian Academy of Sciences, 13 Prospekt Entuziastov, 410049 Saratov, Russia; khlebtsov_b@ibppm.ru

[4] Department of Materials Science, Fudan University, Shanghai 200433, China; solovevlab@gmail.com

[5] School of Engineering and Materials Science, Queen Mary University of London, Mile End Rd, London E1 4NS, UK

* Correspondence: D.Gorin@skoltech.ru

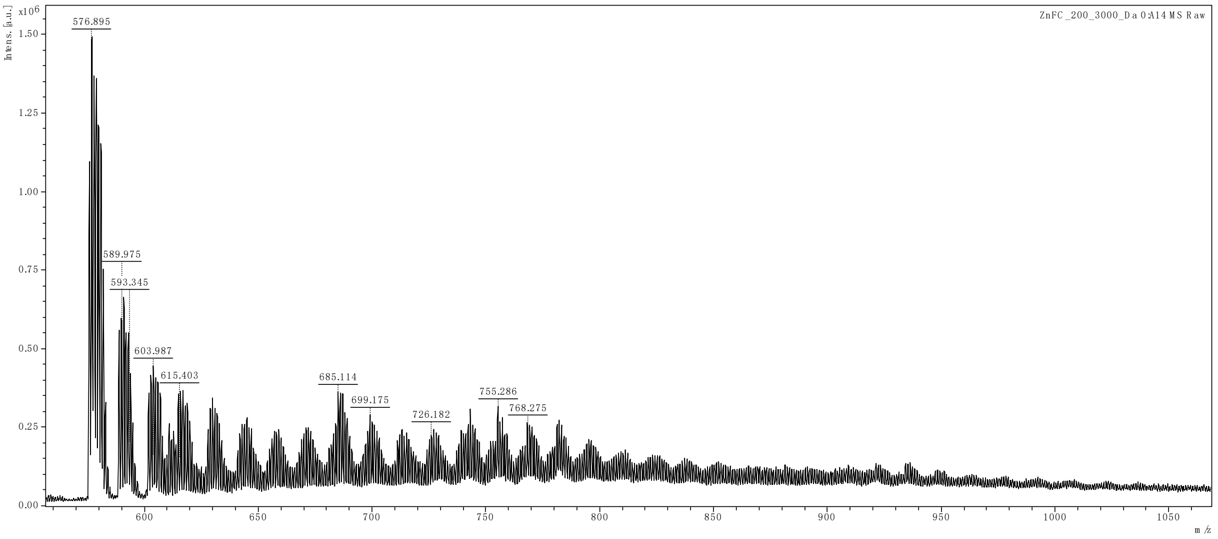

Figure S1. Mass spectrometry measurements for zinc (II) phthalocyanine (ZnPc) used as a dye for microbubbles preparation.

Table S1. Zeta-potential and dynamic light scattering measurements of gold nanoparticles containing samples.

Samples	Zeta-potential measurements (mV)	Mean size obtained with dynamic light scattering measurements (nm)
AuNPs	-49±0.9	15±2
AuNPs coated with BSA	-1±0.4	45±4

Table S2. Zeta-potential measurements of microbubbles-containing samples.

Samples	Zeta-potential measurements (mV)
BSA MBs	-8.4±0.6
BSA-AuNPs MBs	-6.0±0.6
BSA-ZnPc MBs	-6.2±1.0
BSA-ZnPc-AuNPs MBs	-5.3±0.9
BSA-ICG MBs	-6.6±0.9
BSA-ICG-AuNPs MBs	-4.8±0.2

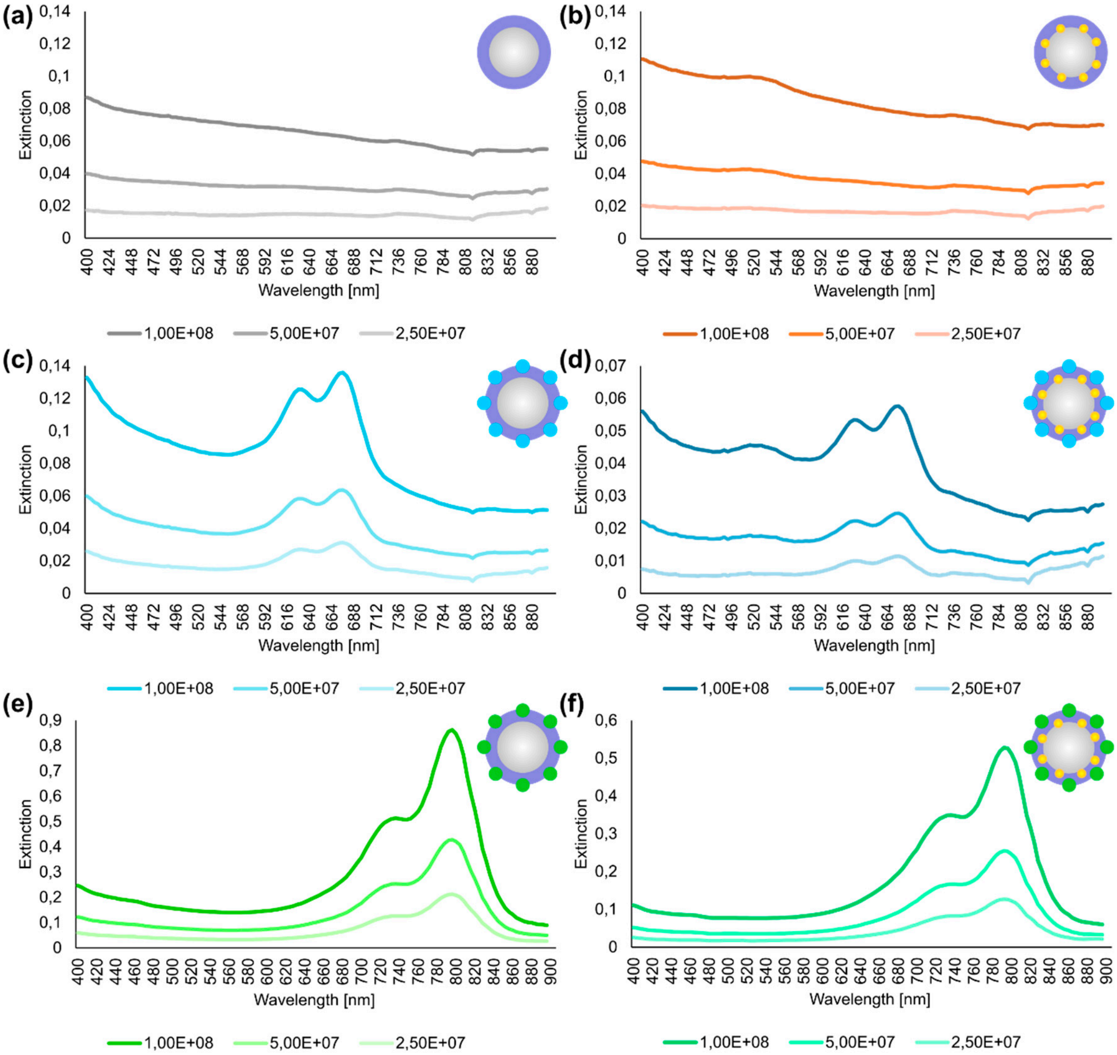

Figure S2. Extinction spectra of obtained bubbles: a) microbubbles with BSA only shell (BSA MBs), b) microbubbles with the BSA shell stabilized with gold nanoparticles (BSA-AuNPs MBs), c) microbubbles with the BSA shell functionalized with ZnPc (BSA-ZnPc MBs), d) microbubbles with the BSA shell functionalized both with ZnPc and gold nanoparticles (BSA-ZnPc-AuNPs MBs), e) microbubbles with the BSA shell functionalized with ICG (BSA-ICG MBs), f) microbubbles with the BSA shell functionalized both with ICG and gold nanoparticles (BSA-ICG-AuNPs MBs). All samples presented in Figure were diluted with concentrations 10^8, $5*10^7$ and $2.5*10^7$ probes / mL.

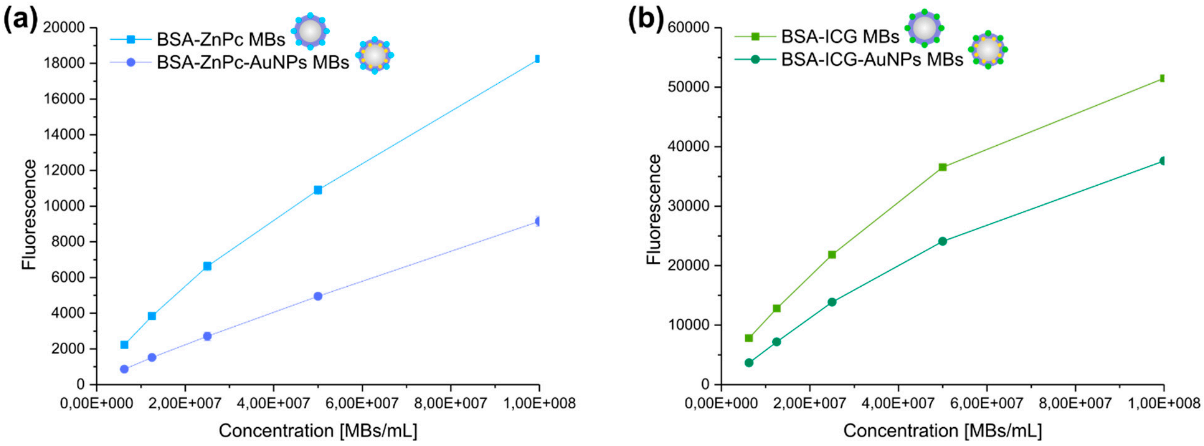

Figure S3. Fluorescence dependence on concentration for obtained microbubbles: a) BSA-ZnPc MBs (light blue line and dots) and BSA-ZnPc-AuNPs MBs (dark blue line and dots) at the wavelength of 700 nm, b) BSA-ICG MBs (light green line and dots) and BSA-ICG-AuNPs MBs (dark green line and dots) at the wavelength of 830 nm. All samples presented in Figure were diluted with concentrations 10^8, $5*10^7$, $2.5*10^7$, $1.25*10^7$ and $6.25*10^6$ microbubbles / mL.
